# The Intraoperative Utility of Raman Spectroscopy for Neurosurgical Oncology

**DOI:** 10.3390/cancers17243920

**Published:** 2025-12-08

**Authors:** Jia-Shu Chen, Jun Yeop Oh, Todd C. Hollon, Shawn L. Hervey-Jumper, Jacob S. Young, Mitchel S. Berger

**Affiliations:** 1 Department of Neurological Surgery, University of California San Francisco, San Francisco, CA 94143, USA; 2 Department of Neurological Surgery, University of Michigan, Ann Arbor, MI 48109, USA

**Keywords:** artificial intelligence, brain tumor, extent of resection, Raman spectroscopy, stimulated Raman histology

## Abstract

Maximal safe surgical resection is a foundational principle in brain tumor surgery. To date, many intraoperative modalities have been developed to help facilitate the identification of brain tumor versus normal brain tissue so that surgical resection is maximized but limited to the boundaries of the tumor for preservation of neurological function. Of note, Raman spectroscopy has been adapted into one of these modalities because of its ability to provide rapid, non-destructive, label-free intraoperative evaluation of tumor borders and molecular classifications and help guide surgical decision-making in real time. In this review, we performed a literature review of the landmark studies incorporating Raman spectroscopy into neurosurgical care to highlight its current applications and limitations. In this modern day, Raman spectroscopy is able to detect tumor cells intraoperatively for primary glial neoplasms, meningiomas, and brain metastases with greater than 90% accuracy. For glioma surgery, a major recent advancement is the ability to detect different mutations intraoperatively, specifically IDH, 1p19q co-deletion, and ATRX, given their implications on survival and how much extent of resection should be ideally achieved. With recent advancements in artificial intelligence and their integration into stimulated Raman histology, many of these tasks can be completed in as fast as ~10 s and on average 2–3 min. Despite the incorporation of artificial intelligence, spectral data can still be heavily influenced by background noise, and its preprocessing has significant variability across platforms, which can impact the accuracy of results. Overall, Raman spectroscopy has significantly changed the intraoperative workflow of brain tumor surgery, and this review highlights the capabilities that neurosurgeons can currently take advantage of in their practice, the existing data to support it, and the areas that researchers can further optimize to improve accuracy and patient outcomes.

## Simple Summary

Raman spectroscopy is an optical technique that measures how the molecular vibrations of chemical bonds change the wavelength of light. In neurosurgery and other medical fields, the Raman effect has been incorporated into a technique called stimulated Raman histology, which maps the quantified energy changes into a pseudo-histologic image that can aid in the identification and diagnosis of different brain tumors. This review discusses the different applications of Raman spectroscopy in neurosurgical oncology, including identification of brain tumor type, grading, and molecular genetics, and how artificial intelligence has augmented its capabilities and usability in recent years. The limitations of Raman spectroscopy are also outlined to help identify areas of future research that can improve and further expand the ways in which Raman spectroscopy can be effectively utilized in neurosurgical oncology.

## 1. Introduction

Maximal safe resection is a well-established guiding principle in the surgical treatment of brain tumors. In both adults and children, residual tumor after surgery is correlated with a worse prognosis across the vast majority of brain tumors, including glioblastoma, high-and low-grade gliomas, brain metastases, and meningiomas [1,2,3]. As a result, many intraoperative adjuncts including neuronavigation, fluorescence-guidance (e.g., 5-aminolevulinic acid), MRI, and ultrasonography have been developed or incorporated into brain tumor surgery to enhance gross visual inspection of the tumor margins and subsequently facilitate safe maximal resection [4,5,6]. However, for glioma surgery in particular, radiographic evaluation of tumor boundaries for surgical decision-making is limited by the diffuse, infiltrative nature of glioma cells that results in microscopic dispersal that is undetectable on conventional imaging modalities [7,8]. Furthermore, image-guided neuronavigation can become unreliable and inaccurate from the ongoing resection, which distorts the actual anatomy from the registered preoperative images. A more detailed comparison and evaluation of the different advantages and limitations of established modalities for determining tumor boundaries intraoperatively is detailed in Table 1. While intraoperative evaluation of resected tumor via cellular staining and microscopic visualization by a trained pathologist provides more definitive assessments of tumor boundaries, routinely doing so is not feasible from a workforce, time, and logistical perspective. Thus, the advent and application of Raman spectroscopy, a sensitive, specific, and efficient technique capable of evaluating the cellular and molecular features of tissue samples and classifying them as their specific tumor pathology, normal brain, or even necrosis, has been invaluable to the field of neurosurgical oncology.

Raman spectroscopy is a biophotonic technique that quantifies the loss or gain of energy emitted from monochromatic light after it interacts with the molecular vibrations of chemical bonds in a sample of interest. The delta in frequency and wavelength, called the Raman effect, is dependent on the chemical composition and structure of the sample that the incident light interacted with. By understanding how different chemical bonds shift the energy of light, the summation of spectral changes in an unknown sample can convey the ratio of different molecules in an unlabeled sample and subsequently identify what type of tissue the sample most likely is. The molecular composition of healthy brain tissue and pathological brain tumor tissue has been determined to be vastly different, which has allowed for Raman spectroscopy to develop an important role in the treatment of brain tumors. Normal brain tissue possesses a high level of lipids while gliomas have a lower lipid-to-protein ratio and higher hemoglobin concentration as first demonstrated by Krafft et al. in 2005 [28,29]. With further research, the molecular distinctions continued to expand over time, even including discrimination across the different tumor grades due to the different and greater density of nucleic acids in higher grade tumors [30]. Eventually, in 2015, the first experience of intraoperative Raman spectroscopy was reported where Jermyn et al. utilized a handheld contact fiber optic probe connected to a near-infrared laser to measure the Raman scattering signal of grade 2–4 gliomas undergoing resection [31]. Not only did the experience identify brain tumor tissue with high sensitivity and specificity (93% and 91%, respectively), it was able to acquire the necessary data rapidly and portably (<0.2 s) as well as with very fine resolution (17 cancer cells/0.0625 mm^2^). This minimizes disruption to the neurosurgical workflow and maximizes the surgeon’s confidence and ability to demarcate tumor boundaries for the extent of resection. The intraoperative use of Raman spectroscopy for brain tumor surgery has continued to expand as it has been utilized successfully in more tumor pathologies, acquired more granular molecular classification ability, evolved into newer, more advanced techniques like stimulated Raman histology (SRH), and integrated with artificial intelligence algorithms that increase its reliability and efficiency. This review evaluated the major landmark studies on Raman spectroscopy in neurosurgery to discuss the current applications in brain tumor surgery as well as its limitations to set the stage for a discussion on how the technique can be further improved and incorporated moving forward.

## 2. Current Applications of Raman Spectroscopy in Brain Tumor Surgery

The earliest and most common application of Raman spectroscopy in neurosurgical oncology was for the treatment of gliomas. As previously mentioned, Jermyn et al. were the first to pioneer its intraoperative use in glioma surgery through a handheld Raman probe that could be held directly against suspected abnormal brain tissue, acquire the Raman spectra, and provide sensitive and specific tumor cell detection within a total acquisition time of 0.2 s [31]. In subsequent studies, Jermyn et al. were able to further demonstrate that Raman spectroscopy has the capacity to guide intraoperative decision-making because of its ability to detect invasive cancer cells up to 3.7 cm beyond the tumor boundary on T1-contrast enhanced MRI and 2.4 cm beyond the boundary on T2 MRI [32]. This was accomplished in conjunction with technical improvements in its detection resolution to 6 cancer cells/0.0625 mm^2^. While there have been no clinical trials to date evaluating the impact of Raman spectroscopy probes on survival outcomes in glioma, there have been multiple studies comparing the detection accuracy of Raman spectroscopy and 5-ALA fluorescence, which showed that Raman spectroscopy has greater sensitivity than 5-ALA and increases the actual resection volume relative to preoperative tumor volume on imaging [26,27]. Interestingly, the prospective comparative study of Raman spectroscopy and 5-ALA by Herta et al. also demonstrated that the accuracy of tumor detection increases by ~10% when both techniques are used in conjunction [27]. At our institution, both modalities are commonly used in tandem in cases of suspected high-grade glioma. Further investigation on the clinical efficacy of Raman spectroscopy in glioma surgery and how to optimize its use with other available adjuncts is required.

In addition to intraoperative tumor detection in glioma surgery, Raman spectroscopy has also been adapted to perform glioma grading and molecular classification with high fidelity using differences in spectral band intensities of proteins and DNA across the types, which has important implications in intraoperative decision-making [33]. In 2019, Livermore et al. were able to predict the IDH mutant status of 62 patients with 91% sensitivity and 95% specificity, as well as 1p/19q co-deletion status with 79% sensitivity and 100% specificity, in a total average time of ~25 min [34]. In 2023, with further advancements by way of SRH and artificial intelligence that will be discussed later in this review, Holland et al. developed DeepGlioma (Ann Arbor, MI, USA), which classified IDH mutant status with 94.7% accuracy, 1p/19q co-deletion status with 94.1% accuracy, and ATRX mutation with 91.0% accuracy [35]. The molecular classification of gliomas has intraoperative utility because of data suggesting that maximizing the extent of resection may not be as critical in 1p/19q co-deleted tumors as opposed to their counterparts where 0.1 cm^3^ of residual tumor in IDH mutant astrocytoma may negatively impact survival [36,37]. This further applies to IDH wildtype versus mutant tumors where some schools of thought suggest that resection of non-contrast enhancing tumor mass is more important in IDH mutant tumors, though this remains an area of debate across different studies that requires further elucidation [2,38]. Overall, balancing the risk of supramaximal resection and postoperative functional morbidity from greater extent of resection is critical [39]. Awareness of the molecular status of the tumor, especially 1p/19q co-deletion, is helpful in determining that balance, and Raman spectroscopy can provide that information in real time. Furthermore, while this has yet to be clinically tested since there are no intraoperative molecular targeting agents currently available (e.g., intraoperative IDH mutation targeting agents), when these are developed and implemented, Raman spectroscopy will be pivotal to their effective administration.

While the vast majority of research and clinical application with Raman spectroscopy has been within the realm of glioma surgery to date, in recent years there has been significant expansion into the other types of brain tumors, most notably including meningiomas and brain metastases. Distinguishing between meningioma and healthy tissue can be difficult because meningiomas can arise from a variety of different locations and in proximity to many different structures including the dura, skull base, cranium, ventricles, sella, orbit, and more. The distinction between meningioma and dura is especially important given that the goal of meningioma surgery is Simpson Grade 1 resection with macroscopic complete tumor removal including affected dura and underlying bone. This can be challenging at the lateral most borders because macroscopically the infiltrated dura is most often indistinguishable from healthy-adjacent dura; however, intraoperative Raman spectroscopy has been shown to be capable of fulfilling that role [40]. In a study by Jelke et al., the authors demonstrated that a handheld Raman spectroscopy acquisition probe and trained classifier was able to label meningioma versus dura in an external validation series with 100% sensitivity and 94% specificity [41]. Furthermore, Raman spectroscopy has also been adapted and advanced to begin distinguishing the different grades of meningiomas, which has significant prognostic value as radical resection has been shown to have the most significant treatment effect on recurrence risk in WHO grade II and III meningiomas [42,43]. Three studies by Morais et al., Zhang et al., and Lilo et al. developed classification algorithms capable of distinguishing between WHO grade I and II meningiomas with accuracy greater than 96% [40,42,44]. However, there is no classification system to date capable of delineating grade III meningiomas, thereby remaining an area of future investigation. The application of Raman spectroscopy to brain metastases also remains an important avenue for further research. Currently, Raman spectroscopy labeling algorithms are quite capable of distinguishing between brain metastases and healthy brain tissue, as well as glioma, intraoperatively, with accuracy rates greater than 90% across multiple studies [45,46,47]. However, there has been limited success determining the primary cancer origin site from Raman spectroscopy, with some hypothesizing that the metastatic migration of primary cancer cells alters the biochemistry of tumor cells across different sites so that they have more similar properties, thereby making the ability to distinguish them more difficult [48]. The combinatorial use of 5-ALA and Raman spectroscopy during surgery for brain metastases could also be an exciting potential area of future investigation since they account for each other’s limitations, with 5-ALA having differential fluorescence across different primary cancers and Raman spectroscopy having more reliable tumor identification in general [49].

Perhaps the most significant advancement in the use of Raman spectroscopy is the development of SRH. Frozen section analysis of intraoperative tissue samples with hematoxylin and eosin (H&E) staining and subsequent evaluation by a neuropathologist is the gold standard for guiding intraoperative decisions. However, this operative workflow is severely limited by the time-intensive nature of freezing, sectioning, and staining protocols as well as the overall availability of a trained neuropathologist. Stimulated Raman histology bypasses these limitations because it rapidly generates a virtual H&E image by linearly mapping the signal intensity obtained from Raman spectroscopy of the sample to the actual chemical concentration in a way that also preserves visual contrast between the lipids, proteins, and nucleic acids in the tissue. Technically speaking, this is accomplished by mapping the Raman shift of the specimen relative to CH_2_ chemical bonds (2845 cm^−1^), which is found in lipids, and CH_3_ chemical bonds (2930 cm^−1^), which are found in nucleic and amino acids. Superimposing this data onto red and blue coloring results in a digital representation of a classic H&E-stained slide. Furthermore, the spatial architectural features of the resected tissue is preserved in this technique, which provides important diagnostic information [50]. Besides the logistical hurdles that it overcomes, SRH also has several advantages related to its image quality whereby it increases the resolution of diagnostic, histologic findings that are poorly visualized on H&E historically including axons, nuclear morphology, lipid droplets, and microvascular proliferation, as well as eliminating artifacts that are common in frozen slide preparations [51]. In comparison to the handheld probes that provide direct feedback, SRH is a non-contact technique that requires additional levels of processing, which does lead to a relatively slower workflow. Additionally, the physical contact with the handheld Raman probes allows for more accurate correlation to anatomic location for the surgeon, whereas SRH requires indirect registration to the neuronavigation system for localization. However, with the advent of artificial intelligence and deep learning algorithms in the past decade, one of the greatest advantages of SRH is the ability to digitize its images, which permits the application of computer vision algorithms that can extract, learn, and identify diagnostic features of the tumor, its molecular biology, and the current extent of resection with high accuracy, confidence, and rapidity.

## 3. Integration of Artificial Intelligence with Raman Spectroscopy

The application of machine learning techniques towards Raman spectroscopy and SRH began with conventional AI algorithms including random forest, support-vector machines, k-nearest neighbors, and multi-level perceptron classifiers. These models have good accuracy and intraoperative utility but are limited by the fact that they are manual feature engineering techniques. This means that the feature extraction algorithm must be physically designed by the programmer, which requires domain-specific knowledge and significant time commitment, thereby making the optimization and implementation of these algorithms a time-consuming, error-prone process that has a limited ceiling in its labeling accuracy [52]. The integration of deep learning models with SRH in lieu of these traditional algorithms was thus a major breakthrough. Unlike traditional models, deep neural networks are trainable feature extractors that can learn and optimize a hierarchy of features iteratively to improve classification ability [52]. Furthermore, deep learning convolutional neural networks are more capable of deconstructing hyperspectral Raman images, eliminating noise and low-resolution signal, and specifically extracting complex spectral and spatial signal for training and classification that would otherwise be imperceivable to the trained human eye or classical machine learning algorithms [53]. These advantages have paved the way for many landmark classification systems that have great potential for routine intraoperative implementation.

The first deep learning-based intraoperative classifier for SRH of brain tumors was described in 2020 [52]. In a prospective clinical trial of 278 patients comparing neuropathologist reads to their convolutional neural network model based on the Google (Google LLC) Inception-ResNet-v2 architecture (Mountain View, CA, USA), Holland et al. showed that their model was non-inferior to neuropathologist interpretation with 94.6% and 93.9%, respectively. When evaluating the learned representations that the model utilized for training, they saw that the deep layers of the model based their classification on nuclear and chromatin morphology, histoarchitecture, and axonal density, suggesting that it was capable of extracting and learning from domain-specific feature representations. Of the 14 cases misclassified by the model, the majority were glial tumors that are less commonly encountered and were not well-represented in the training set, including ependymomas, medulloblastomas, and pilocytic astrocytomas, suggesting that improvements in accuracy can be made with just more training data. Interestingly, there were no overlaps in the misclassified cases between the deep learning model and neuropathologist, and most importantly, the automated workflow of the model was timed to provide a diagnosis within ~2.5 min, as compared to 30 min for the neuropathologist workflow. Overall, the integration of complex deep learning models with SRH was proven to be a worthy adjunct to the diagnostic workflow, and it has continued to improve with molecular classification being demonstrated in 2023 with DeepGlioma and, most recently, even faster processing times (<10 s) for detecting tumor infiltration margins with FastGlioma (Ann Arbor, MI, USA). With DeepGlioma, the authors prioritized the identification of IDH-1 and-2 mutations, 1p/19q co-deletion, and ATRX loss, though it is important to note that the model can be abstracted to an arbitrary number of diagnostic mutations. By using a weakly supervised, patch-based contrastive learning technique, DeepGlioma was able to predict IDH mutations with 94.7%, 1p/19q co-deletion with 94.1%, and ATRX mutation with 91.0% accuracy. The IDH mutation accuracy was particularly impressive given that it surpassed the diagnostic performance of IDH1-R132H immunohistochemistry, which is the gold-standard molecular screening modality. The recent release of FastGlioma is also of particular importance given the magnitude to which it is able to scale down the diagnostic processing time and overall operative duration [25]. This was accomplished by using a visual foundation model, which is the first of its kind in neurosurgical oncology and will allow for FastGlioma to be generalizable to other human cancers given its basis in self-supervised training [54]. Most importantly, when compared to the current standard-of-care adjuncts including image-guided neuronavigation and 5-ALA, FastGioma had a false-negative/tumor miss rate of 3.8% as compared to 24.0% in the latter, a nearly 6-fold decrease in the relative risk of leaving residual tumor in the resection cavity and increasing the risk of tumor recurrence and faster time to mortality. Over the past decade, significant strides have been made in the scientific understanding of Raman spectroscopy, brain tumor biology, and how to integrate the two into a clinically effective tool. With artificial intelligence, the field is entering a new frontier that will be able to translate the aforementioned knowledge base into a cost-effective, widely accessible, and efficacious tool for the treatment of brain tumor and cancer patients everywhere. A summary of the most widely known artificial intelligence pipelines for Raman spectroscopy in neurosurgical oncology to-date is provided in Table 2.

## 4. Disadvantages and Current Limitations of Raman Spectroscopy

While promising, the intraoperative use of Raman spectroscopy still faces several challenges. A major issue is the inherently weak Raman signal, due to the low probability of inelastic scattering, where only a small fraction of incident photons produces the Raman effect. This makes Raman spectroscopy highly susceptible to interference from intrinsic fluorescence and noise from the measurement device or the tissue itself. Additionally, using both fluorescence imaging with exogenous fluorescent agents and Raman spectroscopy can obscure the Raman signal, necessitating careful matching of the excitation and emission wavelengths of both techniques. Generally, this interference can be minimized by shifting the Raman laser to a longer wavelength to avoid exciting fluorescent labels [59]. For instance, recent studies have utilized ex vivo Raman spectroscopy with a 785 nm laser to differentiate glioma from normal brain tissue [26]. This approach also delineated tumor margins in optical tissue phantoms and was compatible with 5-ALA fluorescence agents like verteporfin and temoporfin, which have activation wavelengths around 690 nm and 650 nm, respectively. Furthermore, while increasing laser power and integration times can enhance the signal-to-noise ratio, repeated irradiation may have long-term adverse effects on the in vivo tissue architecture. Therefore, further studies are needed to assess the safety of its use in surgical settings.

Another limitation of Raman spectroscopy is the necessity for extensive preprocessing of spectral data. As mentioned above, background noise can obscure meaningful spectral features, requiring steps such as baseline correction to remove fluorescence background, smoothing to reduce noise, and normalization for consistency across samples. Various methods, including direct and iterative polynomial fitting, mathematical morphology, and neural networks, are employed for preprocessing. However, there is no universal standard for these techniques, as they must be tailored to the specific characteristics of the tumor sample and experimental setup, leading to variability in results and complicating cross-study comparisons. In fact, the spectral appearance of Raman features from brain tumor tissue can vary significantly depending on the preprocessing method used [60]. Currently, fast and reliable preprocessing is essential for in vivo classification of brain tumor tissue using Raman spectroscopy. Such capabilities are still under development and have made significant strides as seen with the recent release of FastGlioma. Again, this highlights the need for sophisticated computational tools like artificial intelligence, and expertise for efficient, accurate, and standardized preprocessing will continue to be a barrier to widespread clinical adoption and consistent application.

## 5. Future Applications

As previously highlighted, Raman spectroscopy has been applied to many different aspects of neurosurgical oncology; however, there are still many other avenues to explore its use. One of the challenges in glioma surgery is differentiating radiation necrosis from residual or recurrent tumor tissue, a critical issue that directly impacts treatment decisions and patient outcomes. Radiation necrosis typically occurs within three years of radiation therapy and appears as an enhancing lesion with surrounding edema on MRI, making it difficult to distinguish from recurrent tumor. Radiation necrosis can be managed conservatively, whereas recurrent gliomas may require aggressive surgical intervention in some cases. Intraoperatively, selecting the area for tissue sampling or identifying margins is challenging, and although pathology is the gold standard for definitive diagnosis, results may be ambiguous due to overlapping histological characteristics. Intraoperative Raman spectroscopy may offer a solution for this surgical dilemma by identifying biochemical changes and Raman signatures associated with malignant transformation versus treatment necrosis that help guide intraoperative decision-making and immediate postoperative treatment. This can be facilitated by developing a comprehensive spectral database cataloging variations between necrotic radiation tissue and recurrent tumor tissues under different clinical conditions. For instance, in a proof-of-concept study, Hollon and colleagues utilized SRH images from 35 patients with suspected recurrent gliomas to train a convolutional neural network and develop an inference algorithm for this issue [61]. The algorithm was trained to identify recurrent gliomas in a retrospective cohort of 48 patients, which included 30 patients with recurrent gliomas and 18 patients with pseudoprogression. Despite the limited patient sample size used to train their convolutional neural network, it achieved a remarkably high diagnostic accuracy of 95.8%, possibly aided by the use of a high number of image patches (over 400,000). However, the need for further external validations remains, given the molecular and spatial heterogeneity of gliomas, which could affect the generalizability and reliability of such diagnostic tools.

Moreover, as new AI-based diagnostic systems emerge for detecting brain tumors in fresh, unprocessed surgical tissue [25,52], it could be beneficial to adapt these diagnostics into more reliable handheld Raman spectroscopy instruments. Integrating the current in vivo Raman spectroscopy devices with AI-diagnostic pipelines would allow for faster, less invasive, and potentially more accurate detection of tumor margins. Currently, the location of where the analyzed tissue sample was resected must be saved on navigation, so there is some potential for error within the multi-step workflow of resection and documenting the corresponding location. However, these handheld systems may require additional AI-training on a more diverse dataset of microscopy fields of view and may be limited by lower precision and sensitivity for molecular analysis. The trade-off between conventional and handheld Raman spectroscopy, which hinges on spectral detail and operational flexibility, needs careful consideration. Whether the immediate identification and improvements in accuracy of tumor margins using handheld Raman spectroscopy outweigh the need for more in-depth molecular analysis should also be evaluated.

Lastly, there is promising potential for Raman spectroscopy to predict the likelihood of tumor recurrence during the initial surgical operation if spectral markers correlated with a tumor’s tendency to recur are identified. By providing a biochemical fingerprint of the tumor tissue, Raman spectroscopy could detect areas prone to malignant transformation or recurrence before they become clinically apparent, which early proof of concept studies have already demonstrated in other cancers [62]. This crucial information would enable neurosurgeons to make informed decisions regarding the extent of tissue removal in real time and tailor immediate post-operative treatments to better manage recurrence. Overall, given the infancy of Raman spectroscopy, its impact on long-term patient outcomes and survival is still unclear and needs to be further investigated. However, this technique’s ability to provide fast and personalized surgical and therapeutic interventions based on each tumor’s unique molecular profile and biology is very promising for its potential to improve patient outcomes.

## 6. Conclusions

In this review, we examine the diverse roles of Raman spectroscopy in neurosurgical oncology, from its origins to its current uses and future potential, emphasizing its critical function in providing essential, real-time, non-destructive feedback on tissue composition for informed surgical decisions. The integration of artificial intelligence is advancing its utility and abilities through significant improvements in diagnostic speed and accuracy. Despite facing challenges such as weak signal intensity and interference from fluorescent agents, the benefits of Raman spectroscopy, particularly its non-invasive nature and ability to provide immediate insights, substantially outweigh these drawbacks. Looking forward, Raman spectroscopy has potential for differentiating between radiation necrosis and residual tumor, predicting tumor progression, and becoming even more widespread across both neurosurgical and non-neurosurgical pathologies. Integrating its findings with other modalities like 5-ALA and imaging techniques could potentially revolutionize and improve the quality of neurosurgical procedures and post-operative care, as well as patient outcomes. Continued research and development are crucial to overcoming its current limitations and expanding the clinical applicability and efficacy of intraoperative Raman spectroscopy for brain tumors.

## Figures and Tables

**Table 1:** Advantages and disadvantages of the most common intraoperative tools in addition to Raman spectroscopy for evaluating brain tumor margins.

Tool	Advantages	Limitations	Comparative Data
Neuronavigation	**Cost** - Affordable technology **Availability** - Ubiquitous across institutions **Processing Time** - Real-time feedback between preoperative imaging and resection cavity	**Tumor Resection Correction** - Does not account for changes in tumor size, shape, and location throughout resection **Interval Assessments** - Multiple interval images of resection progress not possible **Identification of Complications** - Unable to identify active, intraoperative complications	See rows below
Intraoperative Ultrasound (iUS)	**Cost** - Affordable technology **Availability** - Ubiquitous across institutions **Processing Time** - Real-time feedback between imaging and resection cavity **Interval Assessments** - Multiple interval images of resection progress is feasible **Tumor Resection Correction** - Accounts for changes in tumor size, shape, and location throughout resection	**Granularity of Data** - Limited correlation of echogenicity to tumor grade, type, and molecular features - Lower ability to distinguish residual tumor margins **Learning Curve** - Interpreting intraoperative findings requires more experience to guide resection	**Vs. Neuronavigation** - Greater rate of GTR [9,10,11,12,13] - Longer OS [11] - Fewer complications [13] - Better functional status [13] **Vs. iMRI** - Lower rate of GTR [14] - More misinterpreted or undetected tumor [15] - Less expensive [14] - Faster acquisition [15] **Vs. 5-ALA** - More misinterpreted or undetected tumor [12] - Shorter OS or PFS [12]
Intraoperative MRI (iMRI)	**Tumor Resection Correction** - Accounts for changes in tumor size, shape, and location throughout resection **Granularity of Data** - Greater ability to distinguish residual tumor margins **Identification of Complications** - Complete brain imaging allows for earlier identification and intervention of complications	**Processing Time** - Longer operative duration due to image acquisition, patient positioning, and transfer/transport (1 h longer on average) **Interval Assessments** - Multiple interval images of resection progress possible but not logistically feasible given processing time **Cost** - Expensive technology and installation ($3–8 million) - Higher operative costs from longer processing time **Availability** - Predominantly found at tertiary, academic institutions	**Vs. Neuronavigation** - Greater rate of GTR [4,16,17,18,19] - Longer OS or PFS [4,16,19] - Better functional status [4,16,18,19] **Vs. iUS** - See iUS row above **Vs. 5-ALA** - Greater rate of GTR [20,21] - Lower sensitivity [12] - Greater specificity [12]
5-ALA	**Cost** - Affordable **Availability** - FDA-approved for HGG **Processing Time** - Real-time feedback between fluorescence and cavity - Direct visualization of tumor boundary **Interval Assessments** - Multiple interval fluorescence evaluations of resection progress is feasible **Tumor Resection Correction** - Accounts for changes in tumor size, shape, and location throughout resection **Granularity of Data** - Fluorescent signal can correlate to tumor molecular features and histology	**Adverse Medication Effects** - Oral ingestion associated with hypotension, gastrointestinal complaints, transaminitis, and photosensitivity **Applicability** - Limited fluorescence signal in low-grade and non-glial tumors	**Vs. Neuronavigation** - Greater rate of GTR [5,20,22,23,24] - Longer OS or PFS [5,24] **Vs. iUS** - See iUS row above **Vs. iMRI** - See iMRI row above
Raman Spectroscopy	**Processing Time** - Real/near-real time feedback between signal detection and tissue classification - Can be augmented by artificial intelligence **Interval Assessments** - Multiple interval tissue sampling/probing of resection cavity are feasible **Tumor Resection Correction** - Accounts for changes in tumor size, shape, and location throughout resection **Granularity of Data** - Spectroscopy data can be highly predictive of tumor type, molecular features and histologic grading - High spatial resolution increases ability to detect microscopic residual tumor **Applicability** - Technology is very generalizable across different brain tumor pathologies	**Quality of Signal** - Susceptible to interference by fluorescence and background noise - Dependent on good equipment and personnel to extract interpretable spectroscopy data **Cost and Availability** - Financing of proper equipment and personnel makes its use currently limited to select centers and institutions **Tissue Sampling** - Acquisition of data with certain processing pipelines (stimulated Raman spectroscopy) requires tissue samples, which creates risk for injury to the brain	**Vs. Neuronavigation** - Greater accuracy for tumor detection [25] **Vs. 5-ALA** - Greater accuracy and sensitivity for tumor detection [25,26,27]

**Table 2:** Current Raman spectroscopy artificial intelligence pipelines for intraoperative detection of brain tumor resection boundaries.

Pipeline	Device/Wavelength/AI Algorithm	Processing Time	Application	Comparative Data
Jermyn et al., 2015 [55]	**Device**: Handheld contact near-infrared Raman spectroscopy probe attached to laser and spectroscopic detector (785 nm) **Algorithm**: Boosted trees machine learning	0.2 s	Intraoperative classification of Grade II–IV glioma versus normal brain tissue in 17 patients	Accuracy of 92%, sensitivity of 93%, specificity of 91% relative to neurosurgeon’s classification under surgical microscope (73%, 67%, 86%)
Ember et al., 2024 (Sentry System) [47]	**Device**: Handheld contact near-infrared Raman spectroscopy probe attached to laser and spectroscopic detector (785 nm) **Algorithm**: Linear support vector machine	0.1 s	Intraoperative classification of glioblastoma, brain metastasis, or meningioma versus normal brain tissue in 67 patients	Accuracy of 91%, 97%, and 96% for glioblastoma, brain metastasis, and meningioma, respectively
Zhang et al., 2023 [56]	**Device**: Handheld contact visible-resonance Raman spectroscopy probe attached to a portable analyzer (532 nm) **Algorithm**: Principal component analysis-support vector machine	5 s	Intraoperative classification of Grade I-IV glioma versus normal brain tissue in 52 patients	Accuracy of 93%, sensitivity of 97%, specificity of 50% across all grades
Livermore et al., 2019 [34]	**Device**: Renishaw bench-top RA800 series Raman spectrometer (785 nm) **Algorithm**: Principal component analysis-fed linear discriminant analysis	<15 min (including biopsy acquisition)	Intraoperative classification of glioma molecular subtype (IDH-wild type vs. mutant) and tumor type (astrocytoma vs. oligodendroglioma) in 62 patients	Accuracy of 98%, sensitivity of 91%, specificity of 95% for IDH-mutant classification. Accuracy of 92%, sensitivity of 79%, specificity of 100% for oligodendroglioma classification.
Hollon et al., 2018 [57]	**Device**: Standard stimulated Raman spectroscopy microscope with Cell Profiler pipeline for image feature extraction (790 nm) **Algorithm**: Random forest machine learning	Not reported	Classification of pediatric brain tumor versus normal brain tissue, as well as low versus high histologic grading in 33 patients	Accuracy of 97%, sensitivity of 96%, specificity of 90% for lesional tissue classification. Accuracy of 96%, sensitivity of 92%, specificity of 87% for low-grade vs. high-grade classification.
Hollon et al., 2020 (SRH-CNN) [52]	**Device**: NIO stimulated Raman histology imaging system (790 nm) **Algorithm**: Inception-ResNet v2 convolutional neural network deep learning architecture	Not reported	Classification of recurrent glioma, pseudoprogression, or non-diagnostic tissue in an external testing set of 48 patients	Accuracy of 92%, sensitivity of 100%, specificity of 82% for tumor recurrence
Jiang et al., 2022 [58]	**Device**: NIO stimulated Raman histology imaging system (790 nm) **Algorithm**: ResNet50 deep convolutional neural network with supervised contrastive representation learning	<2 min	Classification of skull base tumor versus normal brain tissue in 118 patients	Accuracy of 97% for skull base tumor identification
Hollon et al., 2023 (DeepGlioma) [35]	**Device**: NIO stimulated Raman histology imaging system (790 nm) **Algorithm**: ResNet50 deep convolutional neural network with patch-based contrastive learning	<90 s	Classification of glioma molecular features including IDH mutation, 1p19q-codeletion, and ATRX mutation in 153 patients	Accuracy of 97%, sensitivity of 94%, specificity of 100% relative to IHC (90%, 80%, 100%) for IDH mutant classification Accuracy of 94% for 1p19q-codeletion and 91% for ATRX mutation
Kondepudi et al., 2024 (FastGlioma) [25]	**Device**: NIO stimulated Raman histology imaging system (790 nm) **Algorithm**: Visual foundation model based upon the ResNet34 architecture and a whole-slide transformer for self-supervised learning	<10 s	Classification of diffuse glioma infiltration in 129 patients relative to other surgical adjuncts including neuronavigation and 5-ALA	Accuracy of 98% relative to 76% for neuronavigation and 89% for 5-ALA fluorescence

## Abbreviations

AI	Artificial Intelligence
ALA	Aminolevulinic Acid
ATRX	Alpha-thalassemia Mental Retardation X-linked
IDH	Isocitrate Dehydrogenase
SRH	Stimulated Raman Histology
